# Exploring the Challenges of Adolescent Mothers From Their Life Experiences in the Transition to Motherhood: A Qualitative Study 

**Published:** 2017-09

**Authors:** Massoumeh Mangeli, Masoud Rayyani, Mohammad Ali Cheraghi, Batool Tirgari

**Affiliations:** 1 Nursing Research Center, Kerman University of Medical Sciences, Kerman, Iran; 2 Department of Nursing and Midwifery, Tehran University of Medical Sciences, Tehran, Iran

**Keywords:** Adolescent Mothers, Challenges, Iran, Qualitative Study, Motherhood, Kerman

## Abstract

**Objective:** Early motherhood and its impact on mothers, children, families and communities is a prevalent health challenge in developing countries that needs to be urgently explored. The aim of this study was exploring the challenges encountered by Iranian adolescent mothers during the transition to motherhood.

**Materials and methods:** Inductive conventional content analysis approach was used in this qualitative study. Face to face in-depth semi-structured interviews were conducted with 16 Iranian teenage mothers in the Kerman province of Iran from March to December2016. Data collection continued until the point of data saturation and MAXQDA software was utilized in the analysis of the data.

**Results:** Six main categories increasing burden of responsibility, experiencing physical problems, receiving insufficient support, inefficiency in maternal role, emotional and mental distress; and role conflict and 18 sub-categories were extracted from the data analysis.

**Conclusion:** The findings of this study showed that adolescent mothers experience many physical, psychological, mental and social challenges. Therefore, it is expedient that special attention and care support is made available to them by health care providers. A comprehensive understanding of the challenges encountered by adolescent mothers, will aid the development of culturally appropriate health promotion guidelines and strategie.

## Introduction

Motherhood is a significantly important event in the life of a woman (1). Maternal role attainment is a process that requires acquire necessary abilities, learn appropriate behavior, and establish in maternal identity (2). Preparation to accept the maternal role has important effects on maternal adjustment and transition to adulthood (3). However, increasing number of teenage mothers is one of the important concernsin many countries (4). According to the World Health Organization, every year, approximately 16 million teenage girls give birth worldwide while South Koreahas minimum rate and maximum rate is in sub-Saharan Africa (5). Among 1,000 Iranian adolescent girls, 27 become mothers (6). 

Early motherhood has significantly affected not only adolescent girls, but also their spouse, family, school and the society at large (7). Transition to motherhood need to physical, psychological, social and cognitive preparedness; but teenage mothers are not ready to becoming a mother (8). Motherhood becomes cumbersome and convoluted for teenage mothers, who endure maternal role and developmental task of adolescence simultaneously (3). They must adapt with adulthood social roles, physical changes of puberty, significant brain development, and nurturing of an infant (9). Most of teenage mothers are not in a good socio economic condition so transition to motherhood becomes problematic for them (10).

According to the results of studies, Teen mothers face many physical, psychological, social and spiritual challenges: A constant need for support and training (2), inability to planning and decision making, lack of maternal skills (11) encountering unknown situations and major changes (2), high risk pregnancy and birth (4, 5, 12, 13), mental health problems(depression, anxiety, shock, low self-efficacy, isolation) (8, 12, 14), multiple responsibilities (12, 15), role conflict and identity confusion (12, 16), inadequate social and spiritual support (12), disruption of education and employment (4, 11, 14), financial problems (11), social stigma and, religious or cultural negative reaction (4, 11, 14), inappropriate behavior of health care providers (8), and family conflicts (4).

Low accountability, emotional fluctuations, lack of knowledge and experience, the influence of peers, and high risk behaviors in adolescents; highlights the important role of health care providers (17, 18). In developed countries, early motherhood is be considered asone of important public health issues and is assessed by obstetricians & gynecologists, pediatricians, child psychologists, sociologists, family physicians, and nurses (19). Providing high quality services requires understanding of the needs of teenage mothers, their challenges and capabilities. This goal can be achieved only through a comprehensive qualitative study in different cultures.Exploring teenage mother’s experience of motherhood can generate new insights for policymakers and health care providers resulting in efficient response to the challenges of teenage mothers. This study conducted for exploring the challenges of Iranian adolescent mothers during the transition to motherhood.

## Materials and methods

This qualitative study was conducted through inductive conventional content analysis. 

This study was conducted from March to December 2016 in Kerman, Iran. Kerman is located in southeastern Iran, and has a high rate of teenage mothers. A total of 16 teenage mothers who met the inclusion criteria (being a maximum of 19 years of age at the time of first birth, have a child or children up to 2 years of age, being able to speak Persian, being willing to share personal experiences, living with spouses, not having a history of severe mental illness, and good cooperation with the researcher) participated in this study. Participants were selected purposefully with maximum variation in age, child's age, place of residence (urban or rural), and educational level.

Data was collected through face to face in-depth semi-structured interviews conducted by first author (nursing PhD candidate). The interviews were focused on the experiences of the participants while teenagers were asked to explain their experiences of motherhood in adolescence and their challenges. Central questions were asked and progression was made into specifics, also in accordance with participants' statements additional probing questions were used. The interviews were conducted at specific times and location and lasted for in agreement with the participants45 to 60 minutes, during a 6-month period from March to August 2016. Entire interviews were recorded and transferred into audio files to be entered in the computer and data collection continued until data saturation was reached, when no new information was obtained from the interviews. 

The inductive conventional content analysis approach (Graneheim & Lundeman) was utilized for data analysis. The initial audio files of interviews were listened and recorded interviews there after immediately transcribed verbatim and then read several times to gain a general impression. The resulting text from the interviews was read line- by –line and was broken down into meaningful units (words or sentence or paragraphs), which were then condensed, abstracted, coded, and labeled. Next, the codes were re-read in order to be arranged into categories and sub-categories based on their similarities and differences. The first author performed data coding and all co-authors supervised the coding process. In cases where there is disagreement over the coding, the authors discussed and negotiated the codes until they come to a compromise. Data analysis was done continuously and simultaneously with data collection wherefore the data and the generated codes were constantly compared. MAXQDA10 software was used also for management of data. 

Spending sufficient time for collection and analysis of data, appropriate communication with participants, immersion in the data, member check and peer check, Limiting the baseline review of literature, consideration the opinion of an expert (outside the research team), recording of the research process accurately, and checking results of the study with numbers of similar cases who was not among the participants; were utilized to increase rigor (20).

The Kerman University of Medical Sciences Human Research Committee approved this study (ethics approval number: IR.KMU.REC.1394.591). The study purposes, possible risks, and benefits were explained to the participants. The anonymity of participants, privacy and confidentiality were maintained. Interviews were conducted in a private and non-threatening environment and audio files were kept anonymously in a secure place. Participants were assured of the confidentiality of their responses. Participants were also informed that participation in this study was voluntary and they could withdraw at any time. Written consent was obtained from the participants for the recording of the interviews. During the data collection, the first author was ready to provide help and support; if necessary.

## Results

In this study, participants were 16 adolescents, within the ages of 14 to 18.5 at the time of childbirth. All of them were housewives and had education levels from middle school to diploma. Among most cases their husband were at least 10 years older (Table 1).

Six main categories including increasing burden of responsibility, experiencing physical problems, receiving insufficient support, inefficiency in maternal role, emotional and mental distress, and role conflict and 18 sub-categories were extracted from the data analysis (Table 2).

Most teenage mothers expressed increasing responsibility as one of the main challenges and described it as several responsibilities, lack of time and energy, and restriction on spending for self-interests. Following child birth, teenagers were faced with multitude responsibilities and a sharp increase in workload. Therefore, they experienced physical and mental fatigue and needed help and support from others. Most teens were unable to handle the workload. After becoming a mother, teens have been unable to managing timeand planning. So they did not find the opportunity to consider all things.Many adolescents when encountering various responsibilities of motherhood feel restricted, imprisoned and unable to fulfill self-desires (Table 3, Qutation1-3).

**Table 1:** Demographic characteristics of participants

**Participants**	**Age at ** **childbirt** **h (year)**	**Children ** **age ** **(month)**	**Place ** **of ** **living**	**Current ** **Education level**		**Education ** **level of ** **husband**	**Age ** **difference ** **with husband ** **(year)**			**Job of ** **husband**
1	18.5	6	Rural	Diploma		Bachelor	4			Worker
2	15	24	Rural	Middle school		Diploma	11			Farmer
3	17	6	Urban	High school		Diploma	2			Self employed
4	18	13	Rural	High school		Diploma	14			Worker
5	14	14	Urban	Middle school	University Diploma				10	Engineer
6	17	8	Urban	Diploma	University Diploma			7		Jobholder
7	16	4	Rural	Middle school		Diploma	11			Self employed
8	17	18	Urban	Diploma		Diploma	10			Self employed
9	18	14	Urban	Diploma		Diploma	13			Engineer
10	17	23	Urban	University Diploma		Bachelor	10			Engineer
11	15	10	Rural	High school		High school	7			Self employed
12	17	13	Urban	High school		Diploma	11			Jobholder
13	15	24	Urban	Middle school		Middle school	24			Self employed
14	17	22	Rural	High school		High school	10			Clergyman
15	14	3	Urban	Middle school		Diploma	12			Worker
16	16	24	Urban	Diploma		Diploma	6			Self employed

**Table 2:** Main categories and sub categories of challenges of adolescent mot

**Categories**	**Sub categories**
Increasing burden of responsibility and its outcomes	Several responsibilities in new condition Lack of time and energy Experienced restrictions on spending on personal interests
Physical problems	Health problems related to pregnancy Health problems related to childbirth Health problems related to postpartum and breastfeeding
Receive insufficient support	Received inadequate support from spouse Received inadequate support from family and friends Received inadequate support from health care providers
Ineffectiveness in maternal role	Cognitive incompetency related to essential knowledge for Motherhood Practical skill incompetency in the maternal role
Emotional and mental distress	Fear and worry Regret and frustration Guilt and shame Depression Disruption in relationships of couples
Role conflict	Conflict of maternal and student role Conflict of maternal and adolescent role

Becoming a mother in some teens has been associated with several health problems. These problems could be related to pregnancy, childbirth, postpartum and breastfeeding. According to statements from the participants, some adolescent mothers had experienced hyperemesis gravid arum, eating disorders, anemia, bleeding, and preeclampsia during pregnancy. Most participants had endured a difficult birth, dystocia, disruption of the normal process of childbirth, non-elective cesarean birth, and physical consequences of hard labor. Also, small breast, breast fissures, and mastitis were expressed by the majority of teenage mothers (Table 3, Qutation4-6).

Most teenage mothers needed to receive support because they were faced with new roles, increased responsibilities, health problems, rising costs, and knowledge deficit. Without adequate support they experienced serious challenges in adapting to motherhood.

**Table3:** Quotation of Participants

Quotation 1	*“I had to do all the child care, my baby was crying most of the timeand I had to calm him, I changed * *diapers and washed clothes, I woke up several times at night to breast feed, I was in the hospital when * *my baby was sick, The child was too much work, In addition I have to do housework. I was so * *tired.”*(p12).
Quotation2	*“I do not have free time, I have several responsibilities in life, I am always faced with lack of time, I * *cannot be oblivious to child-rearing and I must consider my child. I devoted all my time to my child; I * *do not have any more time to do further work.”*(p9).
Quotation3	*“Since I became a mother I have become an unhappy person, I cannot go anywhere and do anything, I * *can neither go out for recreation nor travel. I cannot think freely. I mustthink about wife, child and my * *life” *(p10)
Quotation4	*“I was in very bad condition during pregnancy, I was often hospitalizedor I had to rest at home, I also * *suffered from anemia, I was not on a good nutrition plan, I experienced bleeding in pregnancy, my * *doctor said my problem was placenta Previa, I was very swollen, and my blood pressure had risen to * *18. My doctor eventually said I had preeclampsia. I had a seizure at the time of delivery”*. (p4)
Quotation5	*“I had a very difficult birth.The last confirmed date arrived, but labor didn’t start, and I was * *hospitalized for 3 days. When the pain began, I endured it for 15 hours. My doctor said, because your * *age is low and growth is not yet complete, cervical dilatation does not progress”. *(p15)
Quotation6	*“My Nipples were very tiny, it was very difficult for me to give milk to my baby. I had so much pain in * *my breast because it was sore, when the child feeds, I had to clasp my teeth together, to help bear the * *pain”. *(p8)
Quotation7	*“My husband does not help me. He believes that mothers should do all child care. When our child gets sick, * *he says that he does not care. He says the mother must assume responsibility for the care of child” *(p5)*.*
Quotation8	*“I could not rely on anyone, my mother could not get along with me, and I had a bad relationship with * *my mother in law. I have no help from my family, caring for my child was solely my responsibility. We * *had a lot of financial problems, we were tenant, and my husband was unemployed. We had no income * *and the cost for pregnancy and baby care had increased, yet we could not get help” *(p2).
Quotation9	*“I did not know much about the care of children, I expected health care providers to pay more * *attention to me, but they did not. When I asked about depression during counseling I was not given any * *help. They only took statistics of child’s height, weight and my blood pressure. I often needed guidance, * *but they did not answer my questions” *(p7).
Quotation10	*“In most situations, I do not know what to do. I did not know why the baby cries. I had to get help from * *others. I reach out to others who might be able to give good advice. I did not know how to take care of a * *sick child, what to give him medicine or food... Most of the time I had to ask my mother what I should * *do” *(p10).
Quotation11	*“I wanted to breast feed my child but I did not know how to hold the baby and what position I should * *be. My mother took care of me all the time; otherwise, when feeding the baby was suffocated” *(p14).
Quotation12	*“I was always worried, because I thought I could not take care of the kid or nurture him. I think I was * *not capable of doing child care. I felt I was too young for such responsibility. I was convinced I would * *not be successful in caring for child” *(p13).
Quotation13	*“Many times I wanted to go back in time. I regretted it when I was in a very bad state, especially when I * *experienced though times in motherhood. I did not like being a mother. ” *(p3).
Quotation14	*“I had a very negative attitude towards children, especially when I lacked knowledge on what steps to * *take. My baby cries because of my behavior, I do not tolerate children and end up hurting my child” * (p9).
Quotation15	*“I was depressed after childbirth* *. * *I was crying, did not tolerate the crying of my baby. I had no desire * *to get up my own or do something for myself. My relationship with others was reduced and I did not * *want to go out of the house. I was always sad and had a desire to be alone always.”* (p11).
Quotation16	*“When I was pregnant, I and my husband were worried, about occurrences of dangerous problems for * *me or my baby, so we were rarely happy. Sometimes I felt it was my husband's fault in causing early * *pregnancy. I get aggressive whenever I feel inadequate as a mother. Our relationship is not as good as * *before” *(p7).
Quotation17	*“When I became pregnant, the headmaster said should not come to school. She said the presence of a * *student with this condition (swollen abdomen) is not right” *(P3).*”Sometimes, the night before exam, * *when I should have been studying, my baby was sick and I had to leave studying and take care of the * *child” *(p6)*.*
Quotation18	*“Ever since I became a mother, my problems have increased. Usually I like to solve problems alone and * *do not get help from others, but in this case it is not possible. Sometimes I have to break my pride and * *call on others for help”. *(p2)
Quotation19	*“When I go to have fun with my friends, they are uncomfortable that there is a childalso. Child care * *does not allow me to welcome my friends well and stay for a long time besides”*. (p16)
Quotation20	*“When I was in the hospital, nurses taught me and said: “give milk to child in sitting positions because * *breastfeeding in supine position may lead to choking of child” But I believed that nothing will happen* *somost times I breast feed my baby in the supine position”.* (p6)
Quotation21	*“Since I became a mother, I have relegated my personal needs and desires, I no longer place priority * *on shopping, beauty salons and self-gratifying needs. I am more concerned about my child’s welfare at * *all times.”* (P11)

It is evident that they did not receive sufficient support from their wives, families and health care providers. Teenage mothers expected support from their husbands in all child-related responsibilities and this lack of support from spouses was a bitter experience for teenage mothers. Teen mothers were dependent on others and expected their mothers, other family members, friends and school training teams to support them in their role as mothers. This lack of the support has created problems for the teen in the care and upbringing of the child, child-related costs, continuing education and community. Health care providers are the most professional source of support for teenage mothers and are expected to meet their educational and care needs. If health care providers fail to give support to teenage mothers, they would consequently suffer health challenges for them and their child (Table 3, Qutation7-9).

One of the main challenges faced by teenage mothers was ineffectiveness. They lack sufficient knowledge and skills for successful maternal role and therefore depend on others. Many teenage mothers had a knowledge deficit and their information was not enough to take responsibility for maternal and child care. Teenage mothers showed limited skill in relation to motherhood including prenatal care, breast-feeding, caring for children (Table 3, Qutation 10, 11).

Teenage mothers stated that accepting the role of motherhood is associated with emotional and mental distress such as fear and worry, regret and frustration, guilt and shame, depression, and disruption in relationship of couples. Fear and worry was mainly derived from incompetence to accept the responsibilities of motherhood. Teenage mothers also worried about difficult situations in pregnancy and childbirth which were caused by insufficient physical maturity. The regret was associated with unwanted pregnancy, problems related to pregnancy and motherhood, and loss of previous desired position. Incompetence in performing the maternal role developed a sense of guilt and self-blame. Some teenage mothers experienced depression, particularly in the postpartum period. Emotional and physical changes of teenage mothers led to tension and disruption of relationship with partner and family. (Table 3, Qutation 12-16).

Early motherhood causes numerous conflicts in adolescent mothers such as conflict of maternal and student role, and conflict of maternal and adolescent role. Pregnancy and child care results in the inability of teenage mothers to studying and are eventually deprived of education. Early motherhood was in conflict with the special features of adolescence. Although teenage mothers tended to be independent but they had to receive help from others to perform motherhood roles. This causes conflict between independence and dependence in adolescents. Most of teenage mothers were willing to continue their relationship with their friends, but after becoming a mother lost the opportunity of being with friends. Childcare had deprived them of fun with friends and peers. Despite becoming a mother, adolescents were still willing to take risks. They do not understand the need for caution in pregnancy, childbirth and child care. They also do not show interest towards safety and health advice. Some teen mothers struggled between personal need and child care, this resulted in a neglect of them or their child, and the presence of the child disallowed self-centered behaviors (Table 3, Qutation17-21).

## Discussion

This study conducted for exploring the challenges of Iranian adolescent mothers during the transition to motherhood. Increasing burden of responsibility, experiencing physical problems, receiving insufficient support, inefficiency in maternal role, emotional and mental distress, and role conflict were the main challenges of Iranian adolescent mothers.

Increase burden of responsibility was one of the challenges of teenage mothers in this study. It was difficult for teen mothers to meet the multiple needs of child, do housekeeping, going to school, and be present in community alongside friends. Some studies were showed that fatigue and inability have been reported frequently by adolescent mothers and they were not able to establish a good interaction with friends, continue their education and be employed (1, 15). 

Participants also had experienced physical challenges. Physical problems that have been caused by pregnancy, childbirth and breastfeeding; made difficult the maternal role for teenage mothers. Several studies were shown that physical problems are higher in adolescent mothers. Pregnancy and childbirth in adolescence may be associated with preeclampsia, anemia, dystocia, cesarean section, breast fissures, and postpartum fever (21, 22).

Some of teenage mothers in this study did not receive sufficient support. Inadequate support was able to exacerbate the challenges of teenage mothers. Teen mothers needed support on many issues such as child care, financial issues, education, and multiple needs of their own and their children. In Iran, teen pregnancies are the result of legal marriages and often families trying to provide adequate care and financial support for teenage mothers. But sometimes, teenage mothers do not receive adequate support from family, community and health care providers. Several studies showed adolescent mothers do not receive adequate social support (8, 16, 23). Stroble (2013) stated teenage mothers are faced with new expenses related to infant nutrition and care and costs arising from the treatment of mother and child; so had to request financial help from family and friends (24). Wives, mothers, and health care providers are the most important sources of support for teenage mothers and play an important role in life planning and child care (15). 

Ineffectiveness in maternal role was the other problem of teenage mothers. Teenage mothers in this study were not able to take care of the child independently. They had not adequate knowledge and competence. Teen mothers were exposed their children at risk due to knowledge deficit. They might even refer to invalid information resources and aggravate problems. Williamson et al (2013) believed that teenage mothers are not ready to motherhood. They stated that low commitment, inability to change life styles,low accountability, lack of confidence, and dependence on others; show teenage mothers are unpreparedness to maternal role (2).The study of Pogoy (2014) showed that most of teenage mothers acknowledged that they are not able to perform proper care of the child independently (25). know ledge deficit and skills of teenage mothers are about pregnancy and birth, postpartum care, breastfeeding, maternal and child health, child care, contraception, diet, infection control, drug treatment, physical activity, sex, health behaviors, and support resources (26). 

Emotional and mental distress was another problem faced by teenage mothers. They had experienced fear, worry, regret, frustration, guilt, shame, depression, and disruption in relationships of couples.yates (2013) showed that teenage mothers experience fear of inability to accept the maternal responsibilities, shock, depression, denial, fear, shame, regret, loneliness, social isolation, and mental exhaustion (27). adaptation to mothering and adolescence, dependence on others, unwanted pregnancy, poor decision making and problem solving, severe fatigue, unpreparedness, lack of time and energy, repetitive tasks, lack of social support, stigma, and taking responsibility alone are sources of stress for teenage mothers (12). Multiple mental stress can affect the performance and health of the mother and her family adversely (7).

Role Conflict had appeared in numerous forms in adolescent mothers. They were wandering between two worlds. One of these was the conflict between motherhood and being a student. School rules were in conflict with the tasks of motherhood that created a conflict between childcare and meet the expectations of school. Barmao-Kiptanui et al(2015) stated that childcare will prevent teenagers from going to school and sometimes they are forced to drop out of school (23).Participant of this study also faced with the conflict between the roles of mothering and adolescence. They were interested in independence and self-centeredness but in order to meet the child's needs they had to rely on others. They, although were became a mother but tended to take risk and were not interested in caution. Bah (2016) showed that teenage mothers experience serious conflicts between motherhood- image and Self-image (28). Many teenage mothers do not pay attention to prenatal care and refer less to health centers. This can cause low birth weight, iron deficiency anemia and dystocia (29). High-risk behaviors such as substance abuse and smoking are higher among adolescents (17).

When teenage mothers are faced with these challenges, health care providers can help them through several interventions. Health care providers can help teenage mothers to planning and management, requesting help from friends, family and health team to reduce burden of responsibilities (30). Health care providers can assess the physical health of teenage mothers in the prenatal, perinatal and postpartum; and apply appropriate interventions to reduce physical health problems (17). In addition to providing professional care, health care providers must strengthen the role of the family in the support of teenage mothers(7). Psychological and economic support, supportive social environment, providing opportunities for education, counseling, and spiritual support are also other supportive approaches that must be considered by health providers (31). Health care providers can compensate lack of awareness of teenage mothers, provide educational and psychological counseling, identifying and correction of high-risk behaviors, help them to accept responsibility of motherhood, and attract social and familial support to teenage mothers (7, 17). Health care providers can identify stressors in teenage mothers and reduce tensions by guidance on adapting to new role. Health providers can help teenage mothers to learn skills of parenting and child care, and plan for better performance in playing the role of mothers and other roles concurrently (32). 

***Limitations: ***This qualitative study explored the challenges of teenage mothers in only one province of Iran. Therefore, the transferability of findings from this qualitative work should be considered with caution. A comprehensive research requires the inclusion of different provinces in geographical status and cultural and environmental conditions.

## Conclusion

This study explored challenges of Iranian teenage mothers. The results of this study showed that teen mothers are facing many physical, mental, psychological and social challenges. Achieving such results shows that teenage mothers need to the comprehensive support from healthcare providers. Health care providers must help teenage mothers through consultation, education, support, coordination, prevention, and care interventions. Further studies are necessary to explore consequences of early motherhood, and strategies that help teenage mothers to overcome this challenges. However, providing health care for teenage mothers can also result in family and community health.
